# Hamburg Canal as an Odorant and as a Beverage

**Published:** 1871-04

**Authors:** 


### Editorial.

### Hamburg Canal as an Odorant and as a Beverage.

We have taken some pains to find the best authorities on "smells" as influencing the public health, and at length have found an article which answers the object of our search tolerably well. Our readers will find the extract in the Miscellaneous department of this month's Journal, which is all we propose to say on the subject of the smell of our canal, " the pride and glory of the state." The article claims for the Thames the championship of the world as an odorant — the Englishmen probably had not examined our Hâmburg canal. Our remarks upon the subject several months since are fully sustained by the observation of the eminent men we have quoted. In this quotation our citizens are all deeply interested, as it will allow them to breathe easier if they are obliged to go near the perfumed neighborhood. The article referred to does not, however, cover the question of using the products of the dredging of the canal as a condiment in our water. Sewerage water as a beverage does not seem to have suggested itself for investigation. If the Hamburg canal is to be dumped in the river above the inlet to our reservoir, it is but fair that the citizens have due notice, when they will be at liberty to use it or not as they see best. If it is put anywhere in Niagara river, it will of course pass into Lake Ontario, out through the River St. Lawrence and into the Atlantic Ocean, and if increased in strength and efficiency, according to the Homoeopathic principle, by tritruration, both shores of the Atlantic will be unfit for bathing purposes, in a very short time, to say nothing of the waters of the rivers and lakes for culinary purposes. This canal, while it contains no germs of specific disease, in the simple matter of most diabolical smell, appears comparatively harmless; but the *idea* only, that it might *possibly* enter the reservoir and be served in our coffee, and with some, as a slight diluent to stronger beverages, produced a sensation of nausea, and vomiting followed, by violent diarrhoea, causing many families to discard its use altogether. If imagination has done this, pray tell us what will be the effects of the reality.

